# Different Diets in the Same Habitat: How Food Resource Distribution Shapes the Foraging Preferences of Shorebirds in Hangzhou Bay

**DOI:** 10.1002/ece3.72056

**Published:** 2025-09-08

**Authors:** Dingda Chen, Yifei Jia, Kekan Yao, Shengwu Jiao, Lei Jing, Ming Wu

**Affiliations:** ^1^ Wetland Ecosystem Research Station of Hangzhou Bay, State Key Laboratory of Wetland Conservation and Restoration, Research Institute of Subtropical Forestry Chinese Academy of Forestry Hangzhou China; ^2^ School of College of Forestry Central South University of Forestry & Technology Changsha China; ^3^ Center for East Asian‐Australasian Flyway Studies, School of Ecology and Nature Conservation Beijing Forestry University Beijing China; ^4^ Hangzhou Xixi National Wetland Park Ecology & Culture Research Center Wetland Ecosystem Research Station of Xixi Hangzhou China

**Keywords:** food source, foraging preferences, Hangzhou Bay, shorebirds

## Abstract

The Hangzhou Bay wetland is a crucial stopover site along the East Asian–Australasian Flyway. However, as wetland areas decrease and environmental changes occur, waterbirds have to adjust their dietary strategies to adapt to the available resources. In this study, the diet composition of 11 waders in Hangzhou Bay, China, was assessed via stable isotope analysis technology. The results showed that *Gastropoda*, which were at the lowest trophic level of five food sources, dominated the diet of shorebirds due to their large number and easy capture, with an average contribution rate of 32.7%. *Numenius madagascariensis* , *Numenius phaeopus, Arenaria interpres*, and *Anarhynchus leschenaultii* showed a relatively “specialized” foraging preference for *Gastropoda*, with a contribution rate of more than 35%. Other waders showed a more balanced foraging strategy, distributing their feeding more evenly among the five benthic taxa. Foraging preference is not only affected by food supply but also by the physical conditions and survival strategies of waders. For example, the curlews use their slender beaks to probe deep in the sediment, while plovers rely on visual detection to capture prey exposed on the surface of the mudflat. This study highlights the ecological plasticity of shorebird food utilization and emphasizes the importance of optimizing benthic community structure and increasing the supply of highly nutritious food for effective habitat management. These findings provide valuable insights into wetland restoration and shorebird conservation.

## Graphical abstract

Stable isotope analysis of 11 shorebird species in Hangzhou Bay revealed Gastropoda as their primary food source (32.7% average contribution), with four species showing specialized feeding (> 35% reliance) while others displayed balanced strategies, influenced by both food availability and morphological adaptations like bill shape and foraging techniques; these findings underscore the importance of optimizing benthic community structure and nutritious food supply for effective shorebird conservation in changing wetland ecosystems.

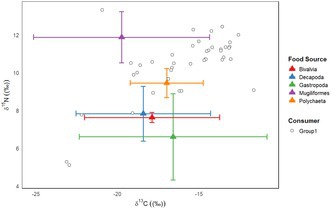

## 1 Introduction

In recent years, as global climate change and human activities have intensified, wetland ecosystems have been facing extensive loss and degradation (Murray 2023). This process directly affects the biodiversity of wetlands, especially bird populations (Wu et al. 2020). As an important ecosystem, coastal wetlands not only provide habitat and food resources for birds but also play a vital role in the global carbon cycle and water purification (Regnier et al. 2022). The survival status of shorebirds is an important indicator of the health status of wetland ecosystems and plays a decisive role in the stability of the regional ecosystem (Amano et al. 2018). However, the reduction and degradation of wetland areas have led to the reduction of shorebirds habitat and food resources, posing a major threat to the survival and migration ability of shorebird populations (Chen et al. 2023). The supply of food resources is crucial for migrating shorebirds, especially on the journey when they often stop to replenish energy, which makes them highly dependent on the food resources of the stopover habitat (Clausen et al. 2018). Therefore, it is important to have an in‐depth understanding of the food source contribution of shorebirds and how the existing food sources in the habitat affect the food source selection of shorebirds. By analyzing the food sources of different shorebird species, revealing their food preferences and niche differences, it is helpful to better understand the functional role of shorebirds in the wetland ecosystem to formulate more effective protection strategies (Pimm et al. 1985).

**FIGURE 1:**
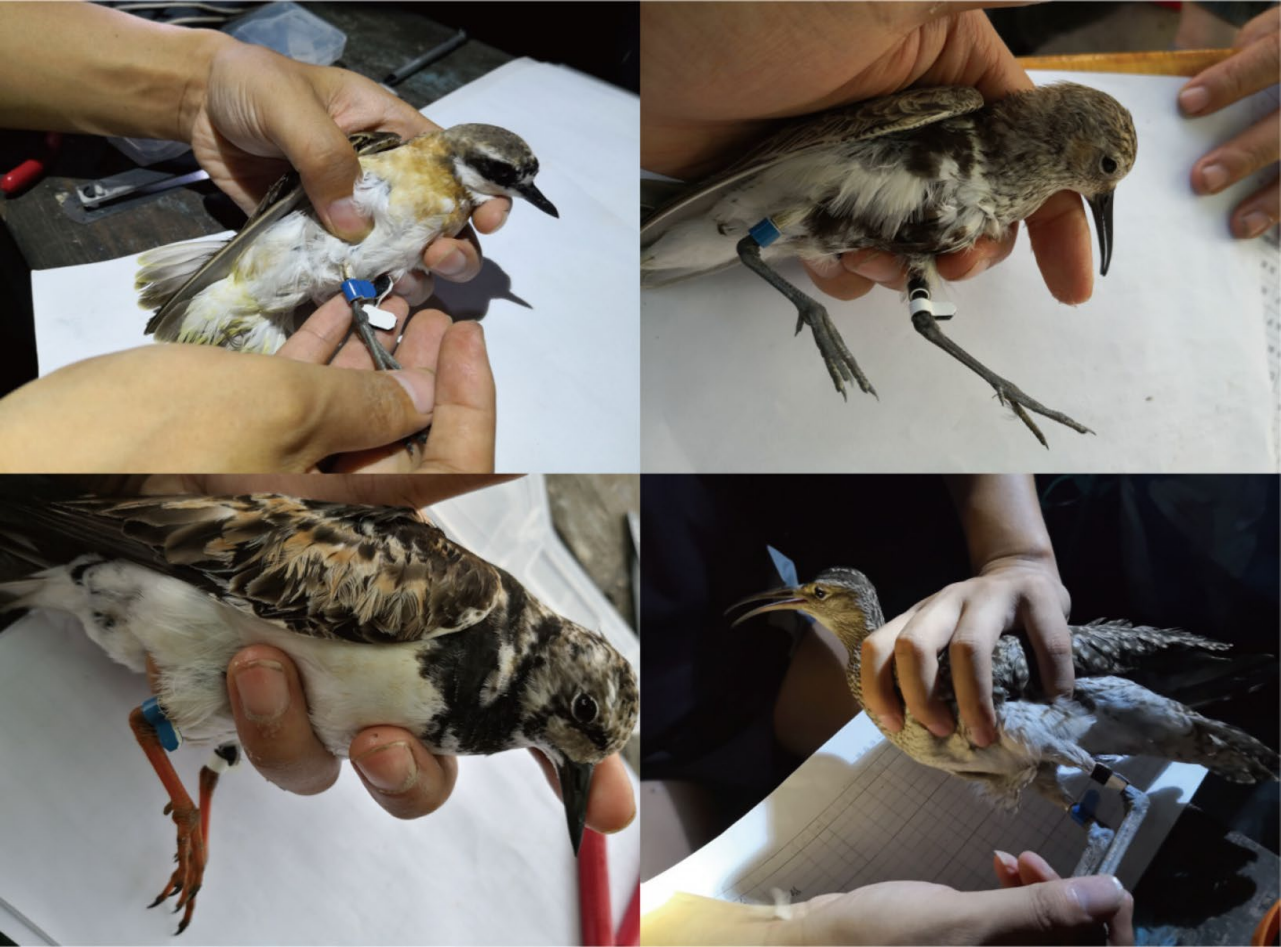
Representative migratory shorebird species photographed during fieldwork in April 2023 in Hangzhou Bay. Clockwise from top left: *Anarhynchus mongolus, Calidris alpina, Numenius phaeopus*, and *Arenaria interpres*. All individuals were documented foraging on the intertidal mudflats during their spring northward migration. These species exemplify the ecological roles of shorebirds utilizing this coastal wetland as a key stopover site. *Photograph by Shengwu Jiao; this photograph is not subject to copyright restrictions and may be used freely for academic publication*.

As shown in Figure 1, several migratory shorebird species—including Anarhynchus mongolus (top left), *Calidris alpina* (top right), *Arenaria interpres* (bottom left), and *Numenius phaeopus* (bottom right)—were observed actively foraging on the intertidal mudflats of Hangzhou Bay during spring migration. These tidal flats serve as critical stopover habitats where shorebirds replenish energy reserves needed for their long‐distance journeys along the East Asian–Australasian Flyway.

**FIGURE 2:**
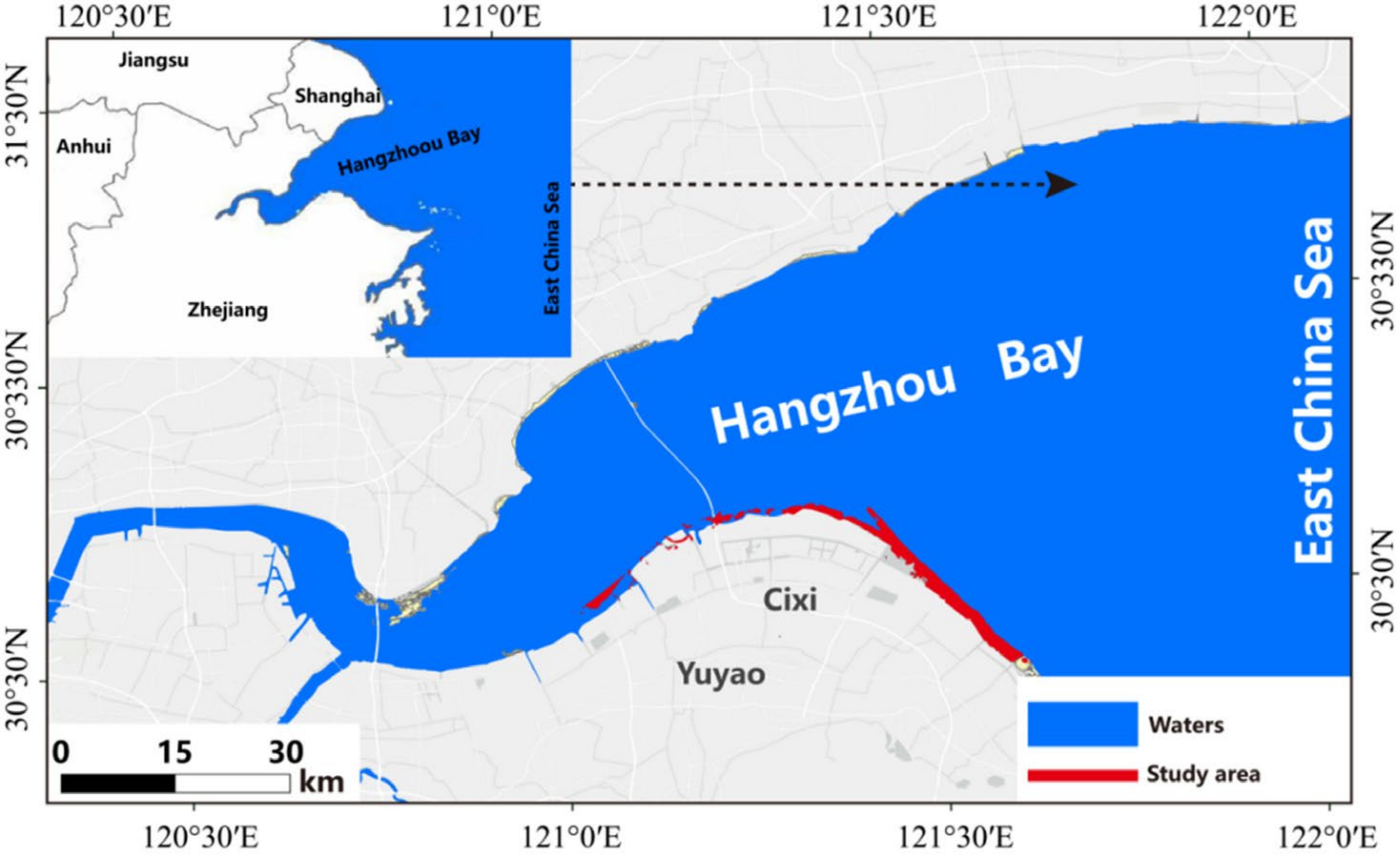
Overview map of the Hangzhou Bay study area.

Located at a key node of the East Asian–Australasian Flyway, Hangzhou Bay is one of the most important stopover and wintering sites for migratory waders along the east coast of China (Figure 2). However, due to the reduction and degradation of wetland areas, increases in human activities, and the invasion of non‐native species, the food resources that shorebirds rely on are facing major challenges. The shortage of food sources is one of the most urgent problems at present and poses a serious threat to the survival and reproduction of shorebirds (He Bo et al. 2023). As an important stopover for migratory birds, the food source structure and quality of Hangzhou Bay Wetland are crucial for providing energy supplements and maintaining bird populations during migration. Despite the abundance of benthos, the lack of high‐quality food resources greatly limits the foraging options of shorebirds (Liu et al. 2024). Shorebirds play an important role in the wetland food web, and their dietary differences reflect their role in energy flow and nutrient cycling (Svendsen et al. 2023; Chatterji et al. 2024). The food choice of shorebirds is not only affected by ecological adaptability but also closely related to the abundance and distribution of food resources in their habitat (Aarif et al. 2025). Therefore, an in‐depth understanding of the food source structure and selection mechanism of shorebirds in Hangzhou Bay will provide theoretical support for wetland ecological protection and shorebirds management and provide a scientific basis for formulating wetland restoration and habitat protection strategies.

Stable isotope analysis has been widely used in the study of bird diet. By measuring the carbon and nitrogen isotope composition of bird tissues (Rogers 2009), the food source and nutritional structure of shorebirds can be accurately identified. This technology records the feeding habits of birds through the “time accumulation effect” of isotopes, and the blood sample reflects the recent food intake (Rubenstein et al. 2002). The application of stable isotope analysis has greatly expanded our understanding of the dynamic changes of bird diet, so that we can comprehensively understand the feeding characteristics and ecological adaptability of wetland birds and provide scientific support for wetland ecosystem management and bird protection. Therefore, this study used a stable isotope analysis method to comprehensively study the food source structure of 11 shorebirds in Hangzhou Bay Wetland, and investigated their food source composition and foraging strategies through blood samples. The results will clarify the ecological mechanism of food source selection of shorebirds in Hangzhou Bay, provide suggestions for wetland protection and management from the perspective of food source structure and foraging strategies, and provide a scientific basis for optimizing Hangzhou Bay Wetland Ecosystem and improving the quality of life of migratory shorebirds.

## 2 Materials and Methods

### 2.1 Study Area

Fieldwork was carried out in April 2023 in the southern intertidal flats of Hangzhou Bay, eastern China. Hangzhou Bay is a critical node along the East Asian–Australasian Flyway and functions as a major stopover site for migratory shorebirds during both spring and autumn migrations. The sampling area is dominated by bare tidal flats, located in the lower intertidal zone with wide, gently sloping surfaces composed primarily of fine mud and silt. These open flats are characterized by minimal vegetation cover, strong tidal influence, high water retention capacity, and long daily exposure durations, making them ideal foraging habitats for shorebirds.

The area supports a high abundance of benthic prey, which serve as critical energy sources for refueling migratory birds. Sampling sites were selected in exposed flats below the high tide line where bird foraging activity was concentrated. All sampling was conducted during early morning low tides to coincide with peak foraging periods. While moderate human disturbance exists, the site retains a relatively intact ecological structure and wetland functionality.

### 2.2 Field Survey and Shorebird Sample Collection

The field survey was conducted in April 2023 during the shorebird migratory season, specifically during the spring northward migration. All 11 shorebird species included in this study are migratory along the East Asian–Australasian Flyway. Most species are considered complete migrants, using the intertidal mudflats of Hangzhou Bay as stopover sites during their spring and autumn migrations for foraging and energy replenishment. Although some of these species have been recorded overwintering along the eastern coast of China, there is no evidence of year‐round residency within our specific study area. Therefore, all individuals sampled in this study were treated as transient migrants, representing typical stopover‐stage resource use. Shorebird capture was conducted during early morning low tides using mist nets placed along the edge of the intertidal zone, targeting individuals actively engaged in foraging. To ensure that the stable isotope signatures in the sampled individuals accurately reflected local dietary sources, several precautions were taken. First, captures were scheduled after the peak of the spring migration period to minimize the inclusion of recently arrived individuals and increase the likelihood that birds had resided locally for several days. Only birds observed foraging before capture were selected for sampling. Whole blood was used as the tissue sample, as its isotopic composition integrates dietary information over approximately 1–2 weeks, providing a temporally representative signal of local foraging activity. Blood samples were collected using minimally invasive venipuncture (Baumans 2005). The area under the humeral vein of the wing was disinfected with an alcohol swab, and a disposable blood collection needle (28 G) was used to puncture the vein. A 20 μL capillary tube was used to repeatedly collect 0.2 mL of venous blood, which was stored in a 1.5 mL blood collection tube. A total of 41 blood samples were collected from healthy shorebirds.

### 2.3 Food Source Survey and Sample Collection

For the food resource survey, ten fixed sampling sites were established along the exposed intertidal mudflats on the southern coast of Hangzhou Bay. The sites were evenly distributed across areas with varying sediment conditions and observed shorebird foraging intensity. At each site, nine replicate quadrats (25 cm × 25 cm; spaced approximately 2–3 m apart) were established, resulting in a total of 90 samples. Sediment within the top 0–20 cm was excavated manually using hand shovels to capture the active vertical range of benthic macroinvertebrates.

Each sediment sample was sieved in the field through a 0.5 mm (500 μm) mesh to remove fine particles and retain larger macrofauna; all retained organisms were carefully sorted under a dissecting microscope, then identified to major taxonomic groups, counted, and weighed (wet mass). This information was used to characterize the community composition and prey availability for shorebirds in the study area. Representative individuals were then oven‐dried at 60°C for 48 h and ground to a fine powder for stable isotope analysis.

Samples were collected by demarcating sample plots and systematically collecting benthic food sources within bird activity areas. At least 3–5 samples were collected from spatially distinct locations for each prey taxon (*Gastropoda, Bivalvia, Decapoda, Mugiliformes*, and *Polychaeta*) to ensure representativeness in stable isotope analysis. Food source samples were soaked and rinsed with deionized water to remove surface debris such as mud and sand. For *Gastropoda*, *Bivalvia*, shrimp, and crabs, the shells were removed, and tissues were collected (Jacob et al. 2005). All samples were acidified by soaking in 1 mol/L hydrochloric acid for 2 h to eliminate the effects of exogenous inorganic carbon on the sample measurements. Afterward, the samples were dried in an oven at 60°C for 8–12 h, thoroughly ground, and passed through a 100‐mesh sieve. The samples were then collected on tin foil and stored in 1.5 mL dry centrifuge tubes.

Since a large number of samples were collected from each food source, typically 4–5 individual samples were mixed to create a composite sample. Five types of food sources were integrated, resulting in 7569 samples (Table 1).

**TABLE 1:** Benthos survey data of Hangzhou Bay.

	*Gastropoda*	*Bivalvia*	*Decapoda*	*Mugiliformes*	*Polychaeta*
Biomass (g)	754.84	128.71	135.83	105	190.25
Quantity	1134	390	61	21	5963
Species	6	4	9	7	7

### 2.4 Sample Preparation for Analysis

All food samples were acidified before analysis to remove interference from carbonates and other inorganic substances (Li et al. 2016). The specific procedure was as follows. After the samples were air‐dried, they were acidified with 1 M HCl. After 24 h, the acid was discarded, and the samples were repeatedly rinsed with distilled water until a neutral pH was achieved. Finally, the samples were dried in an oven at 60°C until a constant weight was achieved.

For blood samples, the serum was separated by centrifugation. To ensure that the blood sample settled at the bottom of the centrifuge tube wall and cap and to prevent blood spillage while ensuring sublimation under vacuum conditions, the top of the centrifuge tube was sealed with a sealing film. Approximately 10 small ventilation holes were created in the film using sterile needles. The samples were then dried in a freeze dryer, and after drying, the blood samples were ground into a powder (Evans Ogden et al. 2004). The powdered samples were stored in dry centrifuge tubes and used directly for isotope analysis.

### 2.5 Stable Isotope Detection

The carbon and nitrogen stable isotope ratios of all samples were analyzed using the Thermo Scientific DELTA V Advantage isotope ratio mass spectrometer. During the analysis, international standards (IAEA‐600, USGS24) were used for calibration to ensure the accuracy of the data.

### 2.6 Statistical Analysis

All statistical analyses were conducted using R version 4.3.1. To assess differences in δ^13^C and δ^15^N values among food source taxa and among shorebird blood samples, we performed one‐way analysis of variance (ANOVA). Where no significant isotopic differences (*p* ≥ 0.05) were detected among sources or samples, those categories were pooled to ensure model parsimony. Isotopic data are presented as means ± standard error (Mean ± SE), and a significance threshold of *p* < 0.05 was applied for all comparisons. Data visualization and further descriptive statistics were carried out using the “ggplot2” and “dplyr” packages.

In the data processing and analysis phases, to ensure the reliability of the research results and to accurately reflect the contribution of each food source to the different bird samples, we employed Stable Isotope Mixing Models (SIMMs) based on the stable isotope model framework for analysis. This model can handle multiple uncertainties and provides robust results. Stable Isotope Mixing Models (SIMMs) have long replaced the widely used stable isotope analysis method in the R (SIAR) package (Qiu et al. 2025). The model infers the dietary proportions of various food sources consumed by organisms by observing the stable isotope values obtained from tissue samples. Given that the running speed of the model was slow and its operation was complex, the data analysis in this study was conducted using the SIMMs package in R.

### 2.7 Selection of Enrichment Factors

The selection of enrichment factors is critical for sample analysis, particularly when studying the isotopic differences between predators and prey within a food chain. Because heavy isotopes are more readily retained in the body, in contrast, light isotopes are more easily metabolized and excreted; the isotope values of predators are generally higher than those of their prey. This difference is known as the trophic enrichment factor (diet–tissue fractionation factor). This value is a key foundation for accurately assessing the composition of food sources. Failure to select appropriate enrichment factors can lead to inaccurate analytical results. In this study, the enrichment factors from related waterbird species were used for the calculation. For blood samples, the enrichment factors used in the study by Wang et al. (2015), who applied stable isotope analysis to avian diet and trophic structure, were adopted. The stable carbon (*Δ*13C) and nitrogen (*Δ*15N) isotope enrichment factors for blood were −0.08 ± 0.38 and 2.60 ± 0.38, respectively (Wang et al. 2015).

### 2.8 Variable Selection

In this study, we collected *δ* ^13^C and *δ* ^15^N isotope data for 33 food sources, which were subsequently grouped and classified based on ecological principles (Liu et al. 2019). The final data were consolidated into five food categories, balancing model simplicity while ensuring the efficiency of the subsequent SIMMs analysis.

Preliminary analysis revealed that the *δ* ^13^C isotope values of the food sources ranged from −12.89% to −28.15%, while the *δ* ^15^N isotope values ranged from 4.17% to 13.53%. After conducting one‐way ANOVA on the *δ* ^13^C and *δ* ^15^N values of all food sources, the results showed no significant differences in *δ* ^13^C isotope values (*F*_4,28_ = 0.77, *p* > 0.05). However, the differences in *δ* ^15^N isotope values were highly significant (*F*_4,28_ = 12.99, *p* < 0.001) (Table 2). This indicates that there are significant differences in *δ* ^15^N isotope values among the food sources, making it appropriate to proceed with the next step of SIMMs analysis.

**TABLE 2:** Mean and standard error of stable isotopes of potential food sources in Hangzhou Bay shorebirds.

Food source and short name	Sample size	Stable isotope signature	
		*δ* ^13^C‰^a^	*δ* ^15^N‰^b^
*Gastropoda*	6	−16.96 ± 5.76A	7.11 ± 2.29A
*Bivalvia*	4	−17.89 ± 4.14A	7.62 ± 0.27AB
*Decapoda*	9	−18.44 ± 4.13A	7.82 ± 1.45AB
*Mugiliformes*	7	−19.76 ± 5.4A	11.87 ± 1.35B
*Polychaeta*	7	−16.91 ± 2.24A	9.67 ± 0.77C

*Note:* Different capital letters following the data indicate significant differences between groups (*p* < 0.01), while the same letter indicates no significant difference (*p* > 0.05).

^a^ There were no significant differences in *δ* ^13^C ‰ values among the food source groups (*F*_4,28_ = 0.53, *p* > 0.05).

^b^ There were highly significant differences in *δ15*N ‰ values among the food source groups (*F*_4,28_ = 13.57, *p* < 0.001).

## 3 Results

### 3.1 Morphological Differences Between Shorebird Groups

Clear morphological differentiation was observed among the 11 shorebird species. One‐way ANOVA revealed statistically significant differences in all measured traits across species. Body mass differed markedly (*F*_10,31_ = 899.79, *p* < 0.001), with larger‐bodied species such as *Numenius madagascariensis* and *Limosa lapponica* weighing substantially more than smaller species like *Charadrius alexandrinus* . Significant interspecific variation was also detected in beak length (*F*_10,31_ = 306.55, *p* < 0.001), head‐beak length (*F*_10,31_ = 357.75, *p* < 0.001), wing length (*F*_10,31_ = 115.67, *p* < 0.001), body length (*F*_10,31_ = 191.22, *p* < 0.001), tail length (*F*_10,31_ = 52.59, *p* < 0.001), and tarsus length (*F*_10,31_ = 181.18, *p* < 0.001). These findings highlight robust morphological divergence among species, likely reflecting ecological specializations in foraging strategy and habitat use (Table 3).

**TABLE 3:** Results of body weight and body part lengths of Shorebirds in Hangzhou Bay.

Body part lengths (mm)								
Species name	Weight (g) mean ± SD	Beak length	Head beak	Wing length	Body length	Tail length	Tarsus	*n*
*Pluvialis squatarola*	205.3 ± 5.9	28.5 ± 1.0	62.3 ± 1.3	174.7 ± 3.1	293.2 ± 4.2	86 ± 2.0	51.5 ± 1.9	2
*Anarhynchus alexandrinus*	49.5 ± 0.7	16.1 ± 0.3	38.5 ± 0.6	109.2 ± 1.1	164.9 ± 0.5	55.3 ± 0.8	29.3 ± 0.7	7
*Anarhynchus mongolus*	74.5 ± 3.1	17 ± 0.6	41.7 ± 0.6	131.4 ± 3.1	189.6 ± 1.6	60.7 ± 1.6	34.6 ± 0.7	2
*Anarhynchus leschenaultii*	87.7 ± 1.6	22.5 ± 0.3	47 ± 0.6	134 ± 1.1	203.5 ± 0.5	60 ± 0.8	37 ± 0.7	5
*Calidris alpina*	52.3 ± 1.6	34.1 ± 0.3	56.6 ± 0.6	116.4 ± 1.1	202.7 ± 0.5	61.2 ± 0.8	27.4 ± 0.7	11
*Arenaria interpres*	118 ± 5.0	18 ± 1.3	41 ± 2.1	145 ± 4.7	215 ± 6.2	67 ± 1.4	25 ± 1.4	2
*Tringa nebularia*	154.1 ± 4.7	51.9 ± 2.1	86.5 ± 2.3	157.4 ± 3.6	334.9 ± 5.6	84.1 ± 1.0	61.7 ± 0.3	4
*Limosa lapponica*	223 ± 13.2	97.5 ± 10.6	144 ± 5.2	228 ± 0.9	405 ± 18.4	77 ± 1.7	60.7 ± 0.7	2
*Numenius phaeopus*	399.5 ± 22.9	79.92 ± 4.3	116 ± 2.0	220.1 ± 30.0	418.1 ± 62.7	106 ± 9.3	64.4 ± 5.1	2
*Numenius madagascariensis*	696.1 ± 28.6	137 ± 7.5	160 ± 5.9	210 ± 7.5	577.5 ± 52.2	116.5 ± 5.2	96 ± 5.8	2

### 3.2 Isotopic Characteristics of Shorebird Blood Samples

After correction for the enrichment factors (Table 4), the average stable carbon isotope value (*δ* ^13^C) of the blood samples from 11 shorebird species was (−16.63 ± 3.24)‰, and the average stable nitrogen isotope value (*δ* ^15^N) was (6.01 ± 1.69)%. Clear interspecific variation was observed in stable isotope signatures among shorebird species. A one‐way ANOVA indicated that both *δ* ^13^C and *δ* ^15^N values differed significantly across species (*δ* ^13^C: *F*_10,30_ = 7.73, *p* < 0.001; δ^15^N: *F*_10,30_ = 6.33, *p* < 0.001). These results demonstrate that different shorebird species occupy distinct isotopic niches, likely reflecting variations in their trophic positions and prey preferences. The significant interspecific differences highlight the ecological diversity within the shorebird community and support the use of stable isotopes in examining dietary differentiation at the species level.

**TABLE 4:** Mean and standard error of stable isotopes in blood samples of shorebirds in Hangzhou Bay.

		Tissues
	Stable isotope signature	Blood (*n* = 41)
*δ* ^13^C/‰	Actual isotope signature	−15.71 ± 3.24 −16.63 ± 3.24 9.85 ± 1.69 6.01 ± 1.69
Corrected using trophic enrichment factors (TEFs)		
*δ* ^15^N/‰	Actual isotope signature	
Corrected using trophic enrichment factors (TEFs)		

### 3.3 Isotopic Differences in Food Sources

**FIGURE 3:**
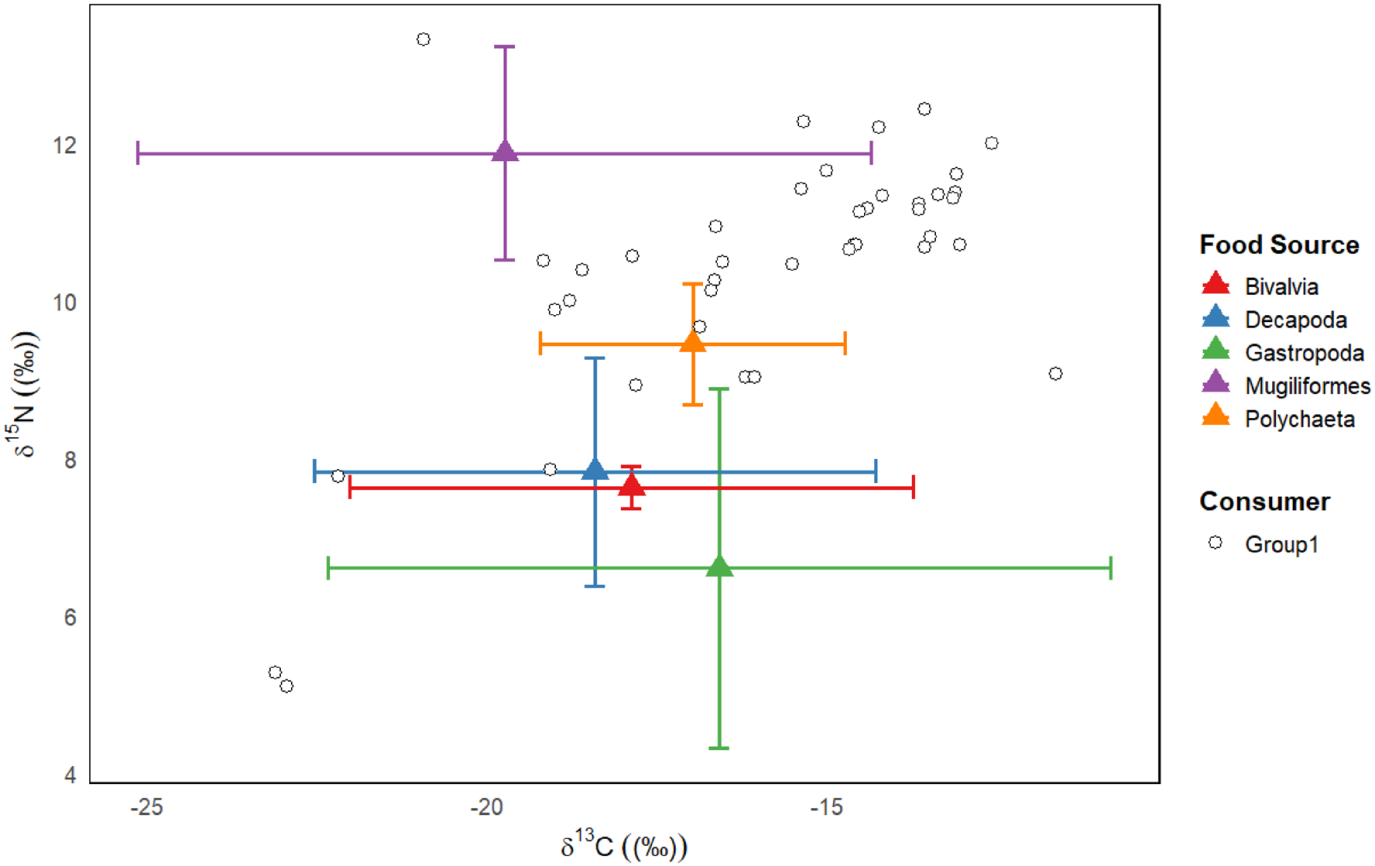
Stable isotope analysis‐food sources and consumers. Bivariate plot of stable carbon (δ^13^C) and nitrogen (δ^15^N) isotope values in Hangzhou Bay. Colored crosses represent the isotopic means (±1 SD) of five food sources—Gastropoda, Bivalvia, Decapoda, Mugiliformes, and Polychaeta. Filled circles (Group 1) denote individual δ^13^C and δ^15^N values derived from the blood of 11 shorebird species sampled in the region.

The average stable carbon isotope values of potential food sources, listed from highest to lowest, are as follows: *Polychaeta* (−16.91‰ ± 2.24‰), *Gastropoda* (−16.96‰ ± 5.76‰), *Bivalvia* (−17.89‰ ± 4.14‰), *Decapoda* (−18.44‰ ± 4.13‰), and *Mugiliformes* (−19.76‰ ± 5.4‰). The average stable nitrogen isotope values of potential food sources, listed from highest to lowest, are as follows: *Mugiliformes* (11.87‰ ± 1.35‰), *Polychaeta* (9.67‰ ± 0.77‰), *Decapoda* (7.82‰ ± 1.45‰), *Bivalvia* (7.62‰ ± 0.27‰), and *Gastropoda* (7.11‰ ± 2.29‰) (Figure 3). *Mugiliformes* exhibited the highest average stable nitrogen isotope (*δ* ^15^N) value, reaching 11.87‰, indicating that these species occupy higher trophic levels in the food chain. *Decapoda* and *Bivalvia* had relatively lower *δ* ^15^N values of 7.82‰ and 7.62‰, respectively, suggesting that species in these categories are generally positioned at intermediate trophic levels. *Gastropoda* exhibited the lowest average *δ* ^15^N value of 7.11‰, indicating that these species are at lower trophic levels in the food chain, typically primary consumers or benthic predators. *Polychaeta* had the highest average stable carbon isotope (*δ* ^13^C) value (−16.91‰), suggesting that these species may predominantly derive from terrestrial or nearshore environments, or that their food webs contain a higher proportion of C3 plants, such as terrestrial plants or algae. In contrast, the *δ* ^13^C value of *Mugiliformes* was the lowest at −19.76‰, which is typically associated with marine or saline environments, suggesting that these species likely inhabit coastal or marine environments. The *δ* ^13^C values of *Bivalvia* and *Decapoda* fall in between, indicating that these species may reside in transitional environments, such as intertidal zones or estuaries, that are influenced by both terrestrial and marine factors.

### 3.4 Average Food Source Composition Reflected by Shorebird Blood Samples

The average contributions of food sources to shorebird blood samples were as follows: *Gastropoda*>*Bivalvia* > *Decapoda*>*Polychaeta* > *Mugiliformes*(Table 5). *Gastropoda* contributed the most to shorebirds, accounting for 32.7%, whereas *Mugiliformes* had the lowest contribution rate at 10.9%. Among the five food sources, *Gastropoda* had the highest contribution, with the smallest contribution exceeding 22%. In particular, the contribution of *Gastropoda* to *Numenius phaeopus* reached over 75%, indicating that it was a major food source for shorebirds. In contrast, the contribution of *Mugiliformes* remained the lowest across all five food sources, which suggests that *Mugiliformes* is not a primary food source for shorebirds, or that it is metabolized and accumulated in smaller quantities within the bodies of birds.

**TABLE 5:** Stable isotope analysis of blood samples of fledglings in Hangzhou Bay shorebirds.

Proportion of each source/%		
Food source	Result based on blood	
	Mean ± SD	95% CI
*Gastropoda*	32.7 ± 16.4	17.5–77.5
*Bivalvia*	21.6 ± 6.5	4.8–21.5
*Decapoda*	21.0 ± 5.2	4.8–20.6
*Mugiliformes*	10.9 ± 2.9	4.0–12.4
*Polychaeta*	14.4 ± 4.2	4.3–14.6

### 3.5 Differences in Food Source Composition Reflected by Shorebird Blood Samples

**FIGURE 4:**
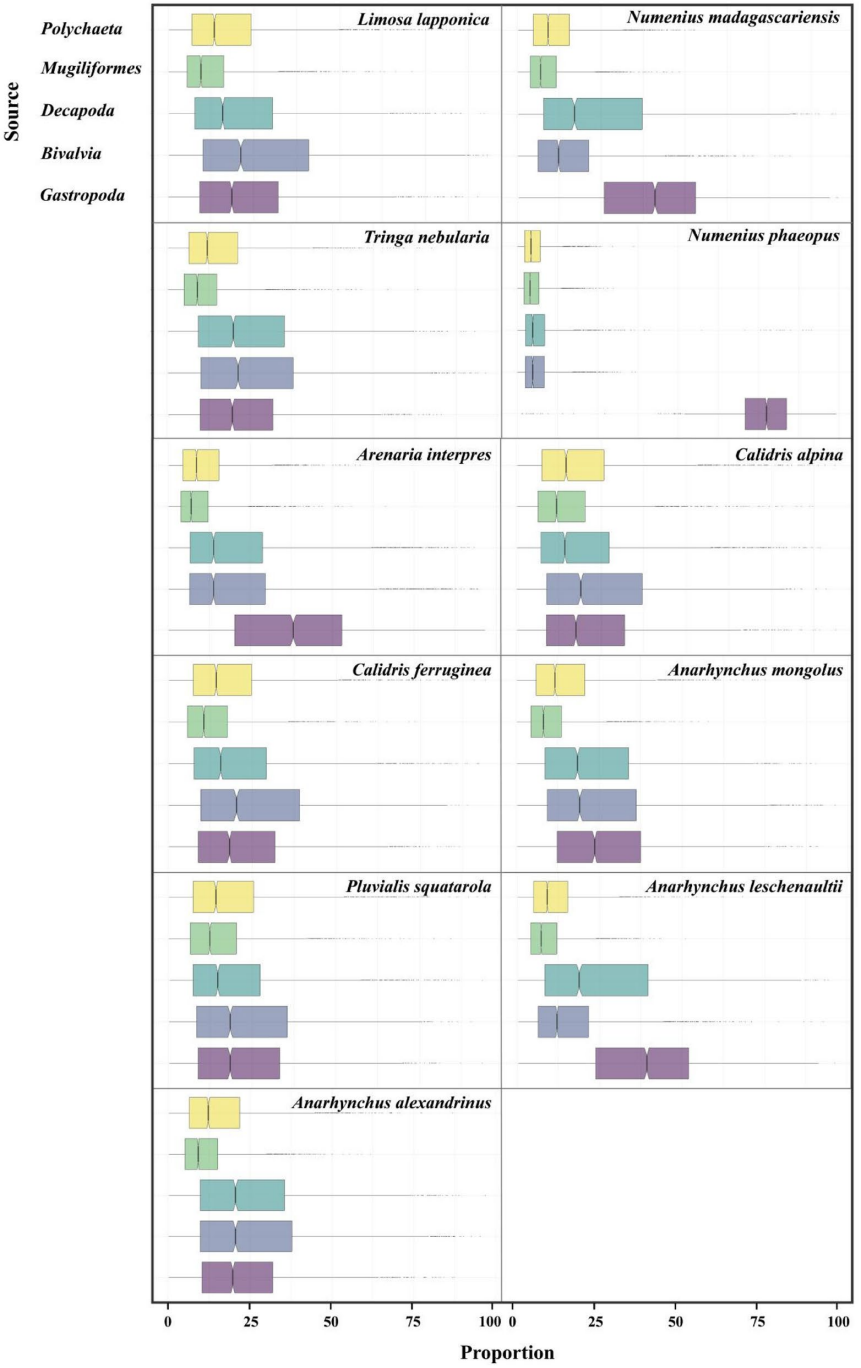
Boxplot of the contribution proportion of food sources for 11 shorebird species in Hangzhou Bay wetland.

Bayesian mixing model results based on blood stable isotope signatures revealed pronounced interspecific variation in food source composition among the 11 shorebird species inhabiting Hangzhou Bay (Table 6; Figure 4). *Numenius phaeopus* exhibited a strong dietary specialization, with *Gastropoda* accounting for 76.7% ± 10.3% of its inferred diet, far exceeding that of any other species. Similarly, *Numenius madagascariensis* (39.6% ± 2.0%) and *Anarhynchus leschenaultii* (38.2% ± 1.9%) also showed a high dependence on *Gastropoda*, suggesting a foraging preference for mollusk‐rich microhabitats. In contrast, several species such as *Limosa lapponica* , *Calidris alpina* , *Calidris ferruginea* , *Pluvialis squatarola* , and *Anarhynchus alexandrinus* exhibited relatively even contributions from all five food sources, indicative of a more generalist foraging strategy. Among these, contributions from *Bivalvia* and *Decapoda* were consistently moderate (e.g., ~24%–27%), while *Polychaeta* and *Mugiliformes* remained secondary sources. Notably, *Arenaria interpres* and *Tringa nebularia* demonstrated intermediate patterns, with *Gastropoda* contributing approximately 22%–37% of their diets, but without a single dominant food type. The contribution of *Mugiliformes* was generally low across species, ranging from 5% in *N. phaeopus* to 15.1% in *P. squatarola* , suggesting limited reliance on fish‐based resources.

**TABLE 6:** Contribution proportion of food sources in blood samples from 11 shorebird species in Hangzhou Bay.

	Contribution proportion% (Mean ± SD)					
	Gastropoda	Bivalvia	Decapoda	Mugiliformes	Polychaeta	*n*
*Pluvialis squatarola*	22.8 ± 1.8	23.5 ± 1.7	20.1 ± 1.8	15.1 ± 1.1	18.5 ± 1.5	2
*Anarhynchus alexandrinus*	22.7 ± 1.6	26 ± 2.0	24.4 ± 1.8	11.2 ± 0.8	15.6 ± 1.2	7
*Anarhynchus mongolus*	26.4 ± 1.7	25.2 ± 2.0	23.3 ± 1.8	10.2 ± 0.8	14.9 ± 1.1	2
*Anarhynchus leschenaultii*	38.2 ± 1.9	15.6 ± 1.2	25.9 ± 2.1	9 ± 0.6	11.2 ± 0.8	5
*Calidris ferruginea*	22.2 ± 1.6	25.9 ± 2.0	20.8 ± 1.7	12.9 ± 1.0	18.2 ± 1.4	11
*Calidris alpina*	22.2 ± 1.7	24.5 ± 1.9	19.5 ± 1.7	14.9 ± 1.2	18.9 ± 1.5	2
*Arenaria interpres*	37.2 ± 2.1	21.6 ± 2.1	20.9 ± 1.9	9.1 ± 0.7	11.2 ± 0.8	4
*Tringa nebularia*	22.6 ± 1.5	26.4 ± 2.0	24.2 ± 1.7	11.3 ± 0.8	15.5 ± 1.2	2
*Limosa lapponica*	22.2 ± 1.6	27.1 ± 2.1	21 ± 1.7	12.2 ± 1.0	17.5 ± 1.4	2
*Numenius phaeopus*	76.7 ± 10.3	6.2 ± 0.5	6.7 ± 0.8	5 ± 0.4	5.3 ± 0.4	2
*Numenius madagascariensis*	39.6 ± 2.0	15.7 ± 1.2	24.7 ± 2.1	8.6 ± 0.6	11.4 ± 0.8	2

## 4 Discussion

### 4.1 Dominance of *Gastropod* Species: Resource Abundance Outweighs Nutritional Quality

In the Hangzhou Bay wetland ecosystem, *Gastropoda* species dominated the food source structure of coastal birds, with an average contribution rate of 32.7%. This phenomenon reveals a core ecological principle: the choice of prey by predators depends on the nutritional quality but is more strongly driven by resource availability, foraging costs, and ecological environment dynamics (Cai et al. 2023; Choi et al. 2014; Ponsero et al. 2011). Although the nutritional level of *Gastropoda* is low, they are the main energy source of shorebird communities (Peng et al. 2023). This result reflects the key role of the optimal foraging strategy in the foraging behavior of shorebirds in Hangzhou Bay.

*Gastropod* species in Hangzhou Bay have obvious advantages in quantity and distribution. Their high density makes them the most accessible food source for shorebirds, and the tidal rhythm further enhances their accessibility (Tong and Wilcove 2020). During low tide, many *Gastropoda* are exposed on the surface of the mudflat, so that water birds can easily obtain food without dredging (Liu et al. 2019). In contrast, benthos with higher nutrient levels, such as *Polychaeta* and *Mugiliformes*, are not only less dense, but also more hidden, resulting in higher foraging costs (McKechnie and Wolf 2010). Therefore, when choosing food sources, waterbirds tend to adopt low‐cost and high‐return foraging strategies, while *Gastropoda* meet this standard.

According to the optimal foraging theory (MacArthur and Pianka 1966), predators tend to choose foods that maximize energy intake and minimize foraging costs. Under this theoretical framework, some shorebirds' foraging strategies in Hangzhou Bay are reflected in their preference for high‐density, easy‐to‐catch *Gastropoda*, rather than crabs or fish, which provide higher energy returns but are more difficult to obtain. Shorebirds preferentially choose prey with a high success rate of capture, avoiding the energy consumption needed to find and deal with benthos that are more difficult to capture. In other words, the high dependence of shorebirds on *Gastropoda* does not necessarily mean a “preference” for this food source. Nevertheless, it is more likely to be an adaptive response to an unbalanced food source structure. In the ecological environment of Hangzhou Bay, the *Gastropoda's* numerical advantage far exceeds its low nutritional value, making it a major energy source.

The high density of *Gastropod* provides a stable food source for shorebirds, but their long‐term dependence on prey with lower nutrient levels may have a series of ecological consequences. The energy density of *Gastropoda* is relatively low, so it is difficult to fully meet the high energy needs of large water birds in the middle of migration (Huang et al. 2022). In the absence of more nutritious food sources, shorebirds may need to consume more energy to maintain their energy (McKechnie and Wolf 2010). However, the increased energy consumption required for foraging may lead to an overall energy imbalance, especially for larger waders. This resource mismatch may further reduce survival during migration. The high dependence of shorebird communities on *Gastropoda* in Hangzhou Bay indicates that the population dynamics of benthos strongly affect the stability of their food chain (Mermillod‐Blondin et al. 2020). If the *Gastropoda* population collapses due to environmental changes, pollution, or other human disturbances, the food chain of shorebirds may experience serious instability. However, species with greater plasticity in the utilization of food sources may be better adapted to this change, whereas species that exclusively eat *Gastropoda* will face greater survival pressure (Ponsero et al. 2011).

### 4.2 Foraging Tendencies and Potential Specialization

Our findings reveal clear interspecific differences in shorebird dietary composition, reflecting diverse foraging strategies shaped by species‐specific ecological and morphological adaptations (Zhang, Ma, et al. 2019). Several species, notably *Numenius phaeopus* , *Limosa lapponica* , *Arenaria interpres* , and *Anarhynchus mongolus*, exhibited strong specialization on gastropods, with *N. phaeopus* displaying the highest dependence (76.7%); others exceeded 35% contribution. This pronounced reliance suggests a consistent trophic preference among these taxa, with gastropods serving as their primary dietary component (Xu et al. 2023). Such dietary specialization likely reflects an optimization of foraging efficiency for prey accessibility and handling time. Gastropods are highly abundant and spatially predictable on intertidal mudflats during low tide, making them an energy‐rich and easily accessible resource for refueling during migration (Zhang et al. 2016). In the case of *L. lapponica* and *N. phaeopus* , this prey selection aligns with their foraging needs and high metabolic demand. In contrast, the pronounced specialization of *A. interpres* and *A. mongolus* may derive from their unique foraging mechanisms: the former is adept at flipping algae and cobbles to uncover prey, while the latter relies on exceptional visual acuity to locate gastropods on exposed sediment surfaces.

Other shorebird species, such as *Calidris alpina* , *T. nebularia* , and *A. alexandrinus* , showed no clear dominance of a single prey type; instead, they adopted a generalist strategy with relatively balanced contributions from Bivalvia, Decapoda, Polychaeta, and Mugiliformes. This trophic flexibility likely confers resilience in environments with high spatiotemporal variation in prey availability. The dynamic nature of tidal wetlands imposes fluctuating constraints on benthic prey distribution (Stewart and Munn 2014), and flexible foragers are better equipped to exploit transient resource windows under such conditions (McGinness et al. 2019). Morphological specialization also appears to drive niche partitioning. For example, shorebirds of the genus *Numenius* possess long decurved bills adapted for deep substrate probing, enabling access to infaunal prey otherwise unavailable to surface feeders. This tactile excavation behavior has been shown to enhance prey detection efficiency in soft sediment environments (Mathot et al. 2018). In contrast, species with shorter bills, such as *P. squatarola* or *A. alexandrinus* , are restricted to surface or near‐surface prey, a limitation that promotes a more opportunistic and diversified foraging repertoire (Twining et al. 2023).

These interspecific differences in diet are further shaped by structural constraints within the benthic community. In Hangzhou Bay, lower trophic‐level invertebrates such as gastropods and polychaetes dominate, while high‐trophic prey remain scarce (Sun et al. 2025). Consequently, trophic specialization is often a passive reflection of prey availability rather than active selectivity. For species like *N. phaeopus* , high dependence on gastropods may represent an adaptive response to limited dietary alternatives rather than ecological preference. Conversely, generalist species buffer against such structural limitations by dispersing foraging pressure across multiple prey types (Zhang, Jiao, et al. 2019). With escalating anthropogenic activity in Hangzhou Bay, alterations in benthic community composition may subtly influence prey availability for shorebirds. While direct causal pathways remain to be confirmed, our findings suggest that under shifting resource regimes, some species may exhibit greater foraging plasticity—potentially increasing their reliance on low‐trophic or non‐traditional prey (Nilsson et al. 2020). If sustained, such behavioral adjustments could carry implications for migratory energetics, habitat use, and long‐term survival.

### 4.3 Environmental Drivers and Adaptive Strategies

In the dynamic intertidal environment of Hangzhou Bay, shorebirds exhibit pronounced niche differentiation in foraging strategies. These differences are shaped not only by morphological traits such as beak length and head structure (Lisney et al. 2013; Mathot et al. 2018), but also by prey accessibility modulated by tidal cycles (Austin et al. 2021; Houlang and Yu 2022). Species with short beaks, relying primarily on vision, target exposed prey like crustaceans and mollusks on the mudflat surface, while those with longer bills engage in tactile foraging, probing sediments to locate buried invertebrates such as worms and bivalves (du Toit et al. 2024; Bourbour et al. 2024). This functional divergence reduces interspecific competition and facilitates resource partitioning within the community (Stewart and Munn 2014).

Tidal rhythms impose further structuring by temporally mediating prey availability. Shorebirds stagger their foraging across tidal phases, a rhythm‐driven niche segregation that enhances individual efficiency while promoting population‐level coexistence (Deboelpaep et al. 2023; Chen 2015). Generalist species such as *Arenaria interpres* , *Pluvialis squatarola* , and *Calidris alpina* exhibited relatively even use of five major prey groups, likely reflecting both anatomical limitations in accessing deeper macrobenthos and ecological adaptation to rapidly shifting prey distributions. Their flexible foraging behavior allows them to track spatiotemporal fluctuations in resource availability across microhabitats, conferring resilience under variable tidal exposure (Nilsson et al. 2020; Clausen et al. 2018).

Moreover, empirical evidence suggests some species respond swiftly to short‐term reductions in specific prey types, underscoring the adaptive value of dietary plasticity in coping with increasing environmental unpredictability along coastal gradients (Lu et al. 2022).

## 5 Management Recommendations

The Hangzhou Bay Wetland is a key node in the East Asia–Asia migratory flyway and has significant ecological value, especially for migratory and resident waterbirds. The area provides abundant benthic resources, offering essential support for energy replenishment and breeding activities of waterbirds.

This study highlights the importance of *Gastropoda* species in the diet of waterbirds, particularly how resource abundance and the ease of foraging determine food source selection. Therefore, it is recommended that fishing bans or restrictions be implemented during the peak breeding and migratory periods of shorebirds, with a focus on protecting *Gastropoda* species to meet the energy requirements of waterbirds. This study also found that high‐trophic‐level benthic organisms are severely lacking in the wetland food source structure. It is recommended that the population density and diversity of high‐trophic‐level organisms be enhanced to better meet the food needs of waterbirds.

The Hangzhou Bay Wetland faces dual challenges from natural and anthropogenic pressures, with reduced habitats and food sources, making it more difficult for waterbirds to survive and migrate. Future research should focus on the relationship between benthic population dynamics and waterbird foraging behavior, the long‐term impact of wetland restoration on waterbird populations, and the effects of climate change on habitats. Further research is required to provide scientific evidence for wetland conservation and waterbird management.

## 6 Methodological Limitations and Future Research Directions

We acknowledge that our current use of bulk δ^13^C and δ^15^N analysis may limit dietary resolution and obscure subtle interspecific or individual differences. Future studies could address these limitations through methodological refinements. Compound‐specific isotope analysis (CSIA) would improve trophic resolution, while integrating molecular techniques such as DNA metabarcoding could provide direct evidence of prey identity. Expanding temporal sampling across tidal cycles and migration stages would capture dietary shifts more comprehensively. Finally, focusing on individual‐level variation may uncover niche differentiation that population means cannot reveal. These approaches will help clarify the mechanisms underlying shorebird foraging strategies in dynamic intertidal systems like Hangzhou Bay.

## Author Contributions

**Dingda Chen:** conceptualization (equal), data curation (equal), formal analysis (equal), methodology (equal), software (equal), validation (equal), visualization (equal), writing – original draft (equal). **Yifei Jia:** formal analysis (equal), methodology (equal), supervision (equal), writing – review and editing (equal). **Shengwu Jiao:** conceptualization (equal), funding acquisition (equal), investigation (equal), methodology (equal), supervision (equal), validation (equal), writing – review and editing (equal). **Kekan Yao:** funding acquisition (equal), investigation (equal). **Lei Jing:** supervision (equal), writing – review and editing (equal). **Ming Wu:** funding acquisition (equal).

## Conflicts of Interest

The authors declare no conflicts of interest.

## Data Availability

Data are available at https://doi.org/10.5061/dryad.n2z34tn8k.
